# Sustaining Community Support for Families Affected by Dementia: The Memory Café Initiative in Rural British Columbia

**DOI:** 10.1002/alz70860_106762

**Published:** 2025-12-23

**Authors:** Donna Benson, Barb Stewart, Eric Li

**Affiliations:** ^1^ DB Foundation for Health Research, Naramata, BC, Canada; ^2^ Medical Arts Health Research, Penticton, BC, Canada; ^3^ University of British Columbia ‐ Okanagan campus, Kelowna, BC, Canada

## Abstract

**Background:**

Community organizations play a crucial role in dementia‐focused programs, yet funding uncertainty remains a challenge. This study examines the Memory Café, a community‐led initiative in rural British Columbia that fosters social engagement, education, and support for individuals with dementia and their caregivers.

Over two years, Medical Arts Research and DB Foundation for Health Research, in partnership with a local university, hosted 10 Memory Café events in rural communities, including Cawston, Keremeos, and Princeton. These events, supported by local government funding, engaged diverse partners—Indigenous leaders, musicians, artists, businesses, and healthcare professionals. Activities included musical performances, calligraphy workshops, Tai Chi, and interactive “memory box” sessions. Keynote speakers, including caregivers with lived experience, provided education and social support.

The initiative catalyzed long‐term community projects, including:

*Forget‐Me‐Not Café (Penticton)*: Inspired a weekly dementia‐friendly café.

*Stretching Group (Keremeos)*: Led by a retired therapist, promoting movement for cognitive health.

*Community Benches (Cawston)*: Installed by a local carpenter to create dementia‐friendly spaces.

**Method:**

The research team employed a community‐based participatory action research (CBPAR) approach, collaborating with local stakeholders to design and implement the Memory Café events. They conducted survey with key community partners, volunteers, and participants to assess the program's impact. as well as to evaluate their experiences and gather feedback for improving future initiatives. Observational data were also collected during the events to document engagement levels and interactions among participants.

**Results:**

Four key sustainability factors were identified:

· *Engaging Community Stakeholders*: Collaborative meetings fostered shared ownership, identifying volunteers and funding sources.

· *Leveraging Local Resources*: Partnerships with businesses and organizations provided funding, venues, and services.

· *Incorporating Community Feedback*: Surveys informed program improvements, increasing engagement.

· *Removing Barriers to Participation*: Transportation support, free events, and sensory‐friendly spaces enhanced accessibility.

**Conclusion:**

The Memory Café model demonstrates a scalable, community‐driven approach to dementia‐friendly initiatives. By fostering partnerships, leveraging resources, and engaging stakeholders, community organizations can build sustainable support systems for individuals with dementia and their caregivers. Future efforts will explore digital engagement and volunteer training to expand impact.

